# More Than a Polyp: AFB-Positive Granulomatous Cecal Polyp Unmasking Pulmonary Tuberculosis

**DOI:** 10.14309/crj.0000000000002397

**Published:** 2026-11-13

**Authors:** Prerna Ashok Kherajani, Avleen Kaur, John Hanna, Christopher M. Curtiss, Mohamed Eisa

**Affiliations:** 1 Department of Internal Medicine, State University of New York Upstate Medical University, Syracuse, NY; 2 Division of Gastroenterology and Hepatology, State University of New York Upstate Medical University, Syracuse, NY; 3 Department of Pathology, State University of New York Upstate Medical University, Syracuse, NY

## CASE REPORT

A 76-year-old man presented with 6–7 months of intermittent painless hematochezia, without abdominal, constitutional, or respiratory symptoms. Hemoglobin was 14.5 g/dL (baseline 15.5). Computed tomography angiography suggested possible rectosigmoid extravasation. Colonoscopy revealed a normal-appearing terminal ileum (not biopsied), a 4 mm cecal polyp with a depressed center (Figure 1), a 4 mm rectosigmoid polyp, and nonbleeding hemorrhoids. Histology of the cecal polyp showed suppurative necrotizing granulomatous inflammation with acid-fast bacilli (Figure 2); the rectosigmoid polyp was a tubular adenoma. His wife, an Indonesian immigrant, was treated for pulmonary tuberculosis (TB) 12 years earlier. Chest radiography showed right upper lobe infiltrates, and induced sputum grew *Mycobacterium tuberculosis* complex. He started directly observed 4-drug therapy.

**Figure 1.**
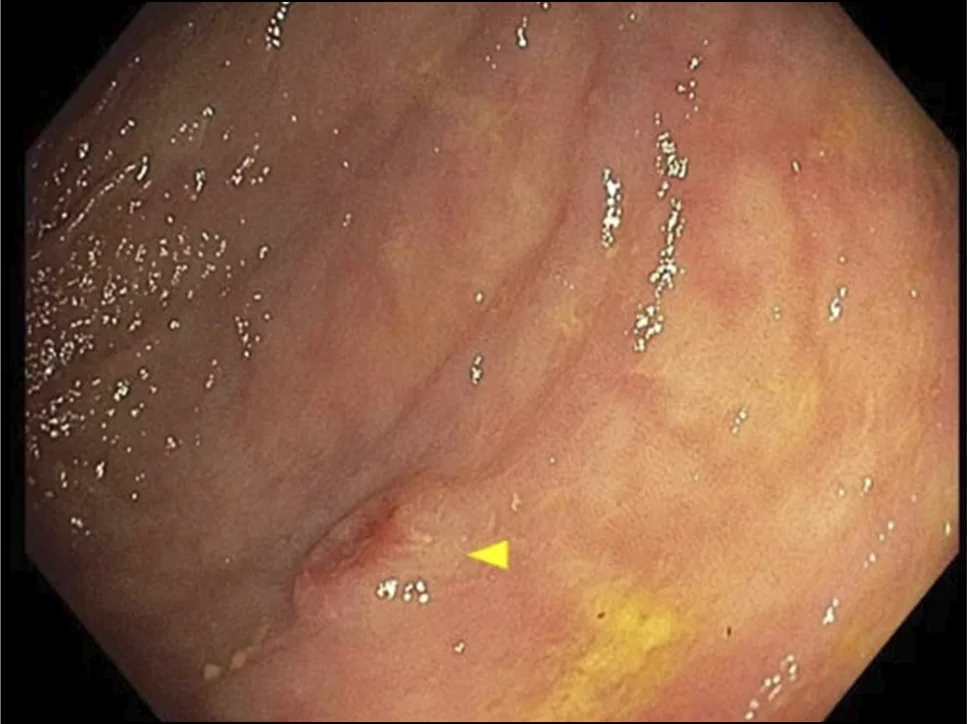
4 mm sessile inflammatory cecal polyp with a depressed center (yellow arrowhead) seen on colonoscopy.

**Figure 2.**
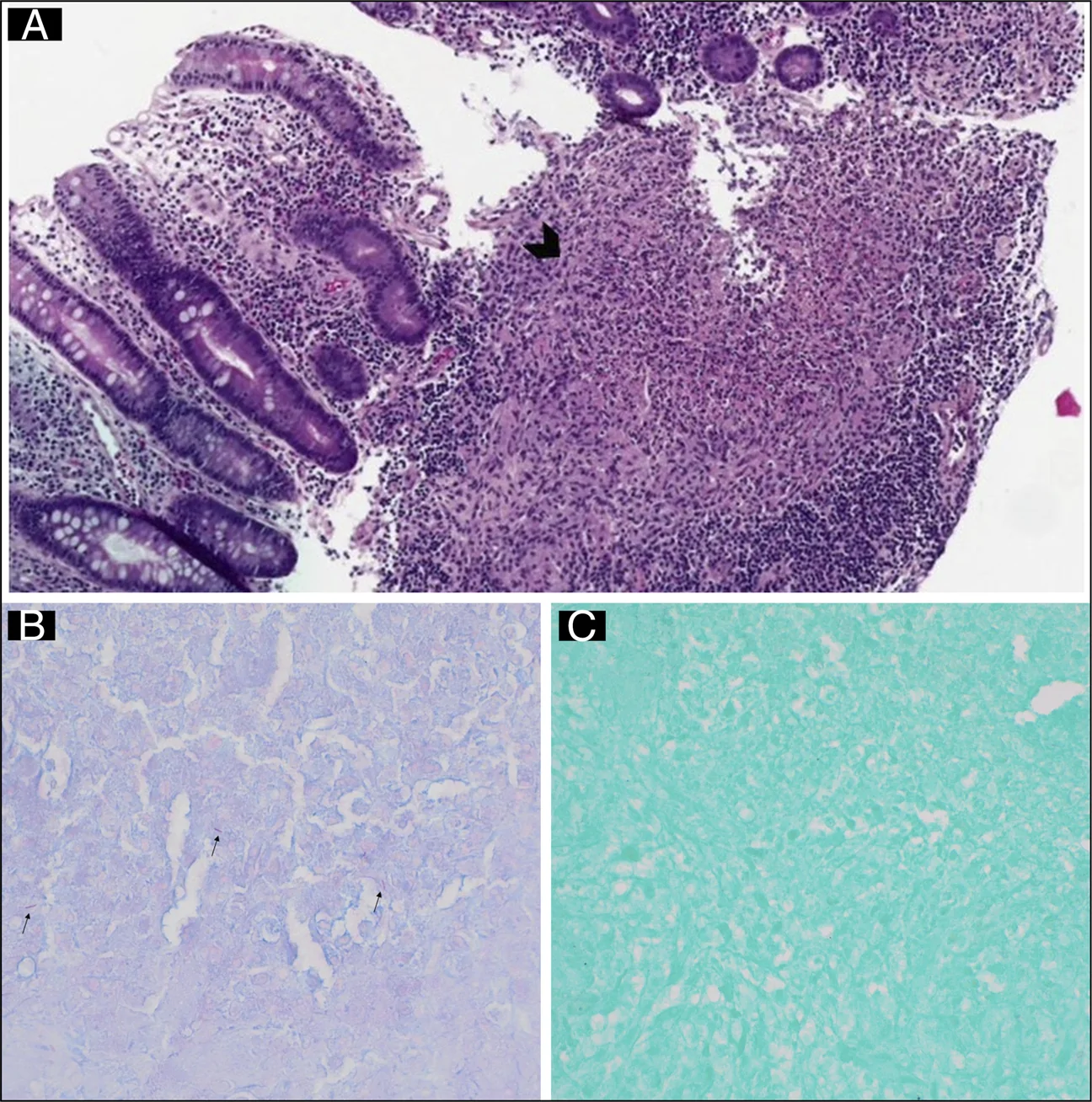
(A) Hematoxylin and eosin stain shows colonic mucosa with focal suppurative necrotizing granulomatous inflammation (arrowhead) and mild active colitis. (B) Acid-fast bacilli stain highlights mycobacteria (arrows) associated with the granulomatous inflammation. (C) Grocott-Gomori methenamine silver stain is negative for fungal organisms.

Hematochezia is usually attributed to benign anorectal disease, diverticulosis, or neoplasia; infectious etiologies are unexpected.^1^ Necrotizing granulomas in colonic tissue have a broad differential, including Crohn's disease, fungal infection, and mycobacteria.^2^ Demonstrating acid-fast bacilli is highly specific but uncommon in tissue.^3^ Here, hemorrhoids likely explained the bleeding, but the polyp's histology prompted an exposure history that revealed household TB contact and led to the diagnosis of pulmonary TB in a patient without respiratory symptoms.^4^ Incidental granulomatous inflammation in a polyp warrants infectious evaluation, especially with exposure risk.

## DISCLOSURES

Author contributions: PA Kherajani and A. Kaur contributed to the conception and drafting of the manuscript. PA Kherajani, A. Kaur, and J. Hanna prepared the images. CM Curtiss and M. Eisa provided critical revisions and supervision. All authors meet ICMJE authorship criteria and approve the final manuscript. M. Eisa is the article guarantor.

Financial disclosure: None to report.

Informed consent was obtained for this case report.
